# Processing–Property Relationships in Fish Protein Hydrolysates: From Controlled Hydrolysis and Peptidomics to Sensory Performance and Translational Readiness

**DOI:** 10.3390/foods15213901

**Published:** 2026-10-30

**Authors:** Nurtay Gubaidullin, Aisarat Gajimuradova, Alfiya Syzdykova, Aibek Zhumalin, Aidyn Igenbayev, Shynaray Zhaskairat, Zhulduz Azhgereeva, Kadyrzhan Makangali

**Affiliations:** 1 Institute of Animal Science and Veterinary Medicine, Saken Seifullin Kazakh Agrotechnical University, Astana 010000, Kazakhstan; nur-tai.kz@mail.ru (N.G.); halik.kz@mail.ru (A.S.); aibek_biotex@mail.ru (A.Z.); 2 Engineering Center for Organic Agricultural Technology, Saken Seifullin Kazakh Agrotechnical University, Astana 010000, Kazakhstan; 3 Institute of Engineering and Food Technology, Saken Seifullin Kazakh Agrotechnical University, Astana 010000, Kazakhstan; a.igenbayev@kazatu.edu.kz (A.I.); sh.zhaskairat@kazatu.edu.kz (S.Z.); z.azhgereeva@kazatu.edu.kz (Z.A.); 4 International School, University of California, 1 Shields Ave, Davis, CA 95616, USA

**Keywords:** fish protein hydrolysates, enzymatic hydrolysis, peptidomics, bioactive peptides, gastrointestinal bioavailability, sensory properties, translational readiness

## Abstract

Fish processing generates protein-rich side streams representing 30–70% of whole-fish mass, creating potential for production of fish protein hydrolysates (FPHs). However, their translation into food or nutraceutical ingredients is limited by variable feedstocks, incomplete process reporting and an evidence base dominated by in vitro assays. This review examines how feedstock origin and pre-analytical quality, controlled enzymatic hydrolysis and downstream processing determine peptide composition and techno-functional, sensory and biological performance. It also evaluates peptidomic workflows and grades evidence from chemical assays through randomized human interventions. Across the reviewed literature, FPHs emerge as heterogeneous, lot-defined products rather than a uniform ingredient class. Maximum degree of hydrolysis is not a universally optimal endpoint: extensive proteolysis may improve solubility and assay responses while reducing protein recovery, interfacial functionality and sensory acceptability. Peptide numbers and candidate sequences depend on liquid chromatography–tandem mass spectrometry (LC–MS/MS) acquisition, database selection, search strategy and false-discovery control. In silico predictions therefore require confirmation with synthetic standards, targeted quantification in the hydrolysate and evaluation of gastrointestinal stability and intestinal transport. Human findings remain limited and inconsistent, precluding class-wide health claims. Translation is further constrained by matrix-dependent acceptable doses and product-specific risks related to contaminants, biogenic amines, microbial and oxidative stability, residual protease activity and allergens. This review proposes minimum reporting requirements and an application-driven translational readiness framework linking feedstock qualification, reproducible processing, molecular identity, biological exposure, matrix compatibility, safety, pilot-scale manufacture and human validation. This approach is required to develop standardized FPHs that deliver prespecified benefits at safe, consumer-acceptable doses while remaining economically and environmentally feasible.

## 1. Introduction

Global fisheries and aquaculture generate large protein-rich side streams, including heads, frames, skin, scales, bones, viscera and trimmings, which may account for 30–70% of whole-fish mass. In 2024, total fisheries and aquaculture production reached 235 million tonnes, including 195 million tonnes of aquatic animals [1,2]. Valorizing these side streams can reduce losses while recovering proteins, lipids, minerals, collagen and enzymes for food and nutraceutical use [3].

Beyond processing side streams, unwanted catches represent an additional potential source of biomass for fish protein hydrolysate (FPH) production. These include non-target fish caught incidentally as by-catch, species of low commercial value and undersized individuals. A proportion of these catches is discarded at sea, with substantial variation among fisheries and fishing gears. For 2010–2014, global marine fisheries discards were estimated at approximately 9.1 million tonnes annually, equivalent to 10.8% of total catch; trawl fisheries were identified as a major contributor [4]. Recovery of suitable unwanted catches could complement the valorization of processing side streams by providing protein ingredients and reducing losses of aquatic biological resources. This opportunity is consistent with the broader contribution of green processing technologies to sustainable food systems discussed by [5], particularly in relation to Sustainable Development Goal (SDG) 2 (Zero Hunger) and S5DG 12 (Responsible Consumption and Production). Its practical contribution depends on lawful retention and use, species identification, rapid stabilization, food safety, affordability and consumer acceptance. Valorization should therefore complement efforts to prevent unwanted catches and improve fishing selectivity [5].

FPHs are heterogeneous mixtures of peptides, free amino acids and residual protein fractions produced by partial or extensive cleavage of fish proteins. Their composition depends on species, anatomical fraction, raw-material freshness, protease specificity, hydrolysis conditions, fractionation and drying [6]. FPHs should therefore be distinguished from fish meal, fish silage, gelatin and purified bioactive peptides, which differ in production route, composition and degree of molecular definition. Enzymatic hydrolysis is generally preferred for food applications because it operates under relatively mild conditions and allows greater control over peptide-bond cleavage and product composition than severe chemical treatments [7]. Industrial relevance, however, depends not only on hydrolysis efficiency but also on enzyme cost, protein recovery, water and energy demand, inactivation, separation, concentration and drying [8].

Degree of hydrolysis (DH) is not a stand-alone measure of product quality. Greater proteolysis can increase low-molecular-weight peptides and solubility while reducing emulsifying or foaming performance and increasing bitterness; enzyme specificity and peptide-size distribution are therefore equally important [9]. Reported antioxidant, antihypertensive, antidiabetic, antimicrobial and anti-inflammatory effects are still dominated by chemical or cell-free assays. Differences in assay conditions, concentrations, controls and molecular fractions limit cross-study comparison, so such results should be treated as screening evidence rather than proof of physiological efficacy [10]. Peptidomics, bioinformatic prediction and molecular docking can prioritize candidate sequences but do not establish activity or exposure. Stronger evidence requires synthetic-peptide confirmation, targeted quantification, gastrointestinal-stability testing and assessment of intestinal transport. Integrated workflows combining hydrolysis, liquid chromatography–mass spectrometry (LC–MS) peptidomics, simulated digestion and Caco-2 (human colorectal adenocarcinoma cell line) monolayer transport have begun to address this gap [11], but human evidence remains limited.

Translation into foods is further constrained by sensory and matrix effects. A systematic review of FPH-fortified foods found that moderate inclusion levels, often around 5–10%, generally gave a better balance between nutritional enrichment and acceptability than higher doses, although the optimum was matrix- and hydrolysate-specific [12]. Bitterness, fishy aroma, colour, texture and lipid stability can therefore limit the deliverable dose. Safety and scale-up must be evaluated together with bioactivity. Relevant controls include feedstock traceability, microbial quality, biogenic amines, heavy metals, lipid oxidation, residual protease activity, allergenicity, storage stability and batch reproducibility. Hydrolysis cannot be assumed to eliminate fish allergens [13], and environmental or economic conclusions depend strongly on processing scale, energy source, drying and co-product allocation [14,15].

**Figure 1:**
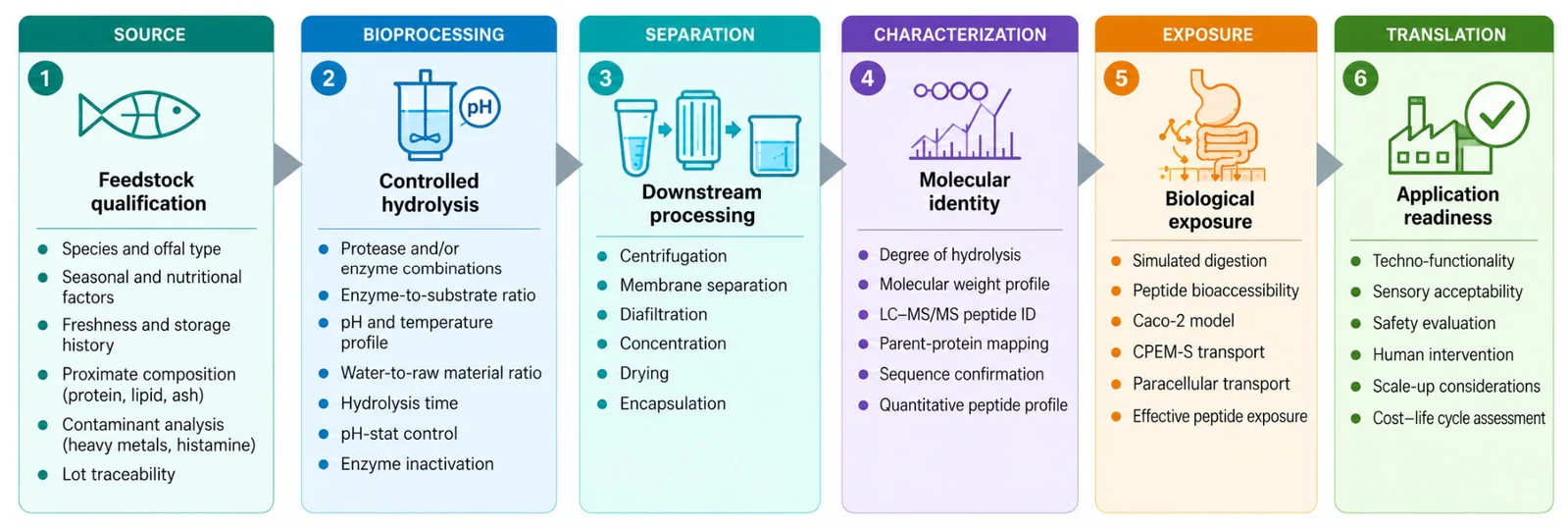
Integrated production-to-application chain shaping fish protein hydrolysate performance. LC–MS/MS, liquid chromatography–tandem mass spectrometry; FDR, false-discovery rate.

This review integrates feedstock quality, controlled bioprocessing, peptidomic identification, gastrointestinal fate, biological validation, sensory performance, safety and industrial feasibility into an application-driven framework. It also proposes minimum reporting requirements and a Fish Protein Hydrolysate Translational Readiness Matrix to distinguish descriptive in vitro activity from evidence supporting reproducible, safe and consumer-acceptable ingredients. The overall production-to-application chain is summarized in Figure 1.

## 2. Feedstock Origin and Pre-Analytical Quality

The broad designation “fish by-products” may refer to materials ranging from muscle-rich trimmings to heterogeneous mixtures of heads, skin, bones, blood, viscera, and intestinal contents. These fractions differ in protein type, collagen, lipid and mineral contents, endogenous enzyme activity, microbial stability, and susceptibility to oxidation. Feedstock should therefore be treated as a lot-defined experimental variable: even when the same enzyme and hydrolysis conditions are used, differences in species, anatomy, storage history, and initial quality can substantially alter peptide composition and the functional and sensory properties of the resulting FPH.

### 2.1. Species, Production System, and Anatomical Fraction

Species-related variation includes differences in muscle structure, collagen content, endogenous proteases, lipid composition, blood retention, and concentrations of pro-oxidant metals. Under standardized hydrolysis conditions, Atlantic salmon, mackerel, and herring heads and backbones produced hydrolysates with distinct sensory profiles. Herring FPHs were generally the most intense and bitter, whereas salmon FPHs were more acceptable. The freeze-dried backbones contained 46.4–51.1% protein, compared with 34.6–38.5% in heads; herring-head ash reached 16.9%, compared with 6.9% in the corresponding backbone. Species had a stronger influence on sensory properties than either anatomical fraction or enzyme. However, fresh salmon and previously frozen pelagic materials were compared, meaning that species and storage effects could not be completely separated [16].

Wild and farmed origin is also relevant but is rarely incorporated into FPH study design. During winter, farmed Brazilian sardine muscle contained 14.52% lipid, compared with 2.37% in wild fish. Wild sardines had a higher relative proportion of long-chain n-3 polyunsaturated fatty acids, whereas farmed fish contained more n-6 fatty acids. Although hydrolysates were not examined, such a sixfold difference in initial lipid content would affect phase separation, oxidative susceptibility, concentration of lipid-derived volatiles, and apparent protein recovery [17]. Anatomical fractions should be defined precisely. Heads combine muscle, connective tissue, bone, eyes, gills, and residual blood; frames contain variable amounts of muscle depending on filleting efficiency; skin is collagen-rich but may retain considerable subcutaneous lipid. Muscle and fillet trimmings consist mainly of myofibrillar and sarcoplasmic proteins and generally produce low-ash, highly soluble hydrolysates. Atlantic salmon muscle hydrolysates, for example, showed protein solubility above 90% under most conditions tested [18]. When species and anatomical fraction vary simultaneously, their effects cannot be separated. Salmon-head hydrolysates contained more glycine and proline, consistent with a greater connective-tissue contribution, whereas Cape hake trimming hydrolysates contained comparatively more glutamic acid, leucine, threonine, and phenylalanine and frequently showed greater activity in chemical bioassays. Because both species and tissue type differed, the source of these differences remained unresolved [19].

Viscera require separate treatment because they contain endogenous digestive enzymes, bile, free amino acids, intestinal microorganisms, and rapidly degradable tissues. Under identical FoodPro PNL hydrolysis conditions, salmon and mackerel viscera yielded more bitter and taste-intense products than heads and backbones. Compounds below 200 Da accounted for approximately 65–75% of visceral hydrolysates, while free amino acids represented about 40 g/100 g of visceral protein. Trimethylamine (TMA), cadaverine, and putrescine were also more abundant, indicating that part of the low-molecular-weight fraction may originate from pre-existing autolysis rather than from the added enzyme alone [20]. Scales are also distinct from skin and frames. Dried red tilapia scales contained 83.9% protein, 15.1% ash, and only 0.9% lipid, representing a collagen- and mineral-rich substrate that requires adequate size reduction, hydration, and enzyme accessibility [21]. In industrially mixed side streams, the proportions of species and anatomical fractions are often unknown. A recent study processed sea bream and sea bass residues containing skin, heads, bones, viscera, and damaged whole fish. The starting material contained 53.2% oil, 41.9% protein, and 7.4% ash, but the proportions of the individual components were not reported, limiting attribution of peptide composition and bioactivity to a particular tissue or species [22].

### 2.2. Time–Temperature History Before Hydrolysis

The interval between fish processing and feedstock stabilization affects endogenous proteolysis, microbial metabolism, lipid oxidation, and protein modification. Whole tilapia waste stored at 4 ± 2 °C for 0, 24, or 48 h and subsequently hydrolysed with the same pepsin treatment produced hydrolysates with different peptide-size distributions and functional properties. Material processed without storage showed greater 2,2-diphenyl-1-picrylhydrazyl (DPPH) radical-scavenging activity, reducing power, and inhibition of linoleic acid oxidation, whereas 48 h storage improved emulsion and foam stability [23]. By contrast, cod, cusk, haddock, and saithe heads stored at −18 °C for up to six months showed only limited changes in hydrolysate yield and proximate composition. However, stable protein, lipid, and ash values do not confirm the stability of peptide sequences, oxidation products, or volatile compounds unless these parameters are measured separately [24]. Recent work with Atlantic herring described mincing through a 4.5 mm plate, preparation of 1 kg batches, immediate freezing at −80 °C, and thawing for 24 h at 4 ± 1 °C. Nevertheless, the catch-to-processing interval, frozen-storage duration, and proportions of heads, backbones, bones, and fillet cuts were not specified [11]. A complete time–temperature record should include the intervals from catch or slaughter to filleting, chilling, freezing, thawing, and hydrolysis, together with material temperature, storage duration, packaging conditions, and the number of freeze–thaw cycles.

### 2.3. Proximate Composition, Oxidation, and Defatting

Moisture, crude protein, total lipid, and ash should be determined in each feedstock lot before hydrolysis and reported on both wet- and dry-matter bases. The protein method and nitrogen-to-protein conversion factor should also be specified. Analysis of only the final dried FPH is insufficient because centrifugation, lipid separation, removal of insoluble material, and drying can conceal differences in the original substrate. Initial lipid content should be considered together with oxidative status. At least one primary oxidation marker, such as peroxide value (PV) or conjugated dienes, should be combined with a secondary marker, such as thiobarbituric acid reactive substances (TBARS), p-anisidine value, or volatile aldehydes. Protein carbonyls are additionally informative for lipid-rich or extensively stored materials. In rainbow-trout heads subjected to induced oxidation before hydrolysis, soluble protein decreased from approximately 83.5% in control FPH to 62.6% in oxidized FPH, which also contained 5.14 nmol carbonyls/mg protein. These results demonstrate that pre-existing oxidation can reduce enzyme accessibility and soluble-protein recovery [25].

Preliminary defatting can reduce the lipid load, oxidation risk, and fishy odour, but its effect depends on the procedure. Mild thermal separation at 40 °C removed up to 85% of the oil from salmon backbones and produced oil with a peroxide value of 0.2 meq/kg and 0.16% free fatty acids [26]. In fatty sockeye-salmon skin, repeated disc-stack centrifugation followed by two isopropanol extraction cycles reduced fat below the detection limit and lowered peroxide value, TBARS, and fishy odour [27]. Defatting does not necessarily improve every FPH property. In Protamex hydrolysates from salmon backbones, thermal oil separation had little effect on molecular-weight distribution, while the non-defatted hydrolysate showed comparatively high DPPH-scavenging activity [28]. Defatting studies should report treatment temperature, centrifugation conditions, solvent-to-material ratio, extraction cycles, antioxidant use, residual solvent, residual lipid, and oxidation indices.

### 2.4. Seasonal Variation and Lot Traceability

Seasonal changes in feeding and reproductive status can alter the composition of raw material. In Baltic sprat, protein content remained relatively stable across autumn, winter, and spring, whereas lipid decreased from 19.2 g/100 g in autumn to 12.9 and 12.1 g/100 g in winter and spring, respectively [29]. Seasonal effects are not uniform across species and tissues. In gurnard by-products hydrolysed with Alcalase under standardized conditions, season had little influence on most FPH properties, whereas anatomical fraction produced clearer differences: the degree of hydrolysis was 24.0–26.5% for heads and 19.0–23.6% for skin–bone mixtures [30]. Because feedstock origin, anatomical composition, cold-chain history and baseline quality are rarely reported consistently, the minimum information that should accompany each FPH study is summarized in Table 1.

Independent feedstock lots should be distinguished from technical replicates. Repeated analyses or hydrolyses of aliquots from one homogenized batch characterize analytical or processing repeatability but do not account for biological, seasonal, or supply-chain variation.

## 3. Controlled Hydrolysis

Controlled hydrolysis should be optimized for a defined application rather than for maximum DH. DH quantifies the average extent of peptide-bond cleavage but does not specify peptide sequence, protein recovery, bitterness or techno-functional performance; the practical endpoint is therefore the peptide profile that meets the intended function at acceptable yield, sensory quality and cost.

### 3.1. Protease Selection and Activity-Equivalent Dosing

**Figure 2:**
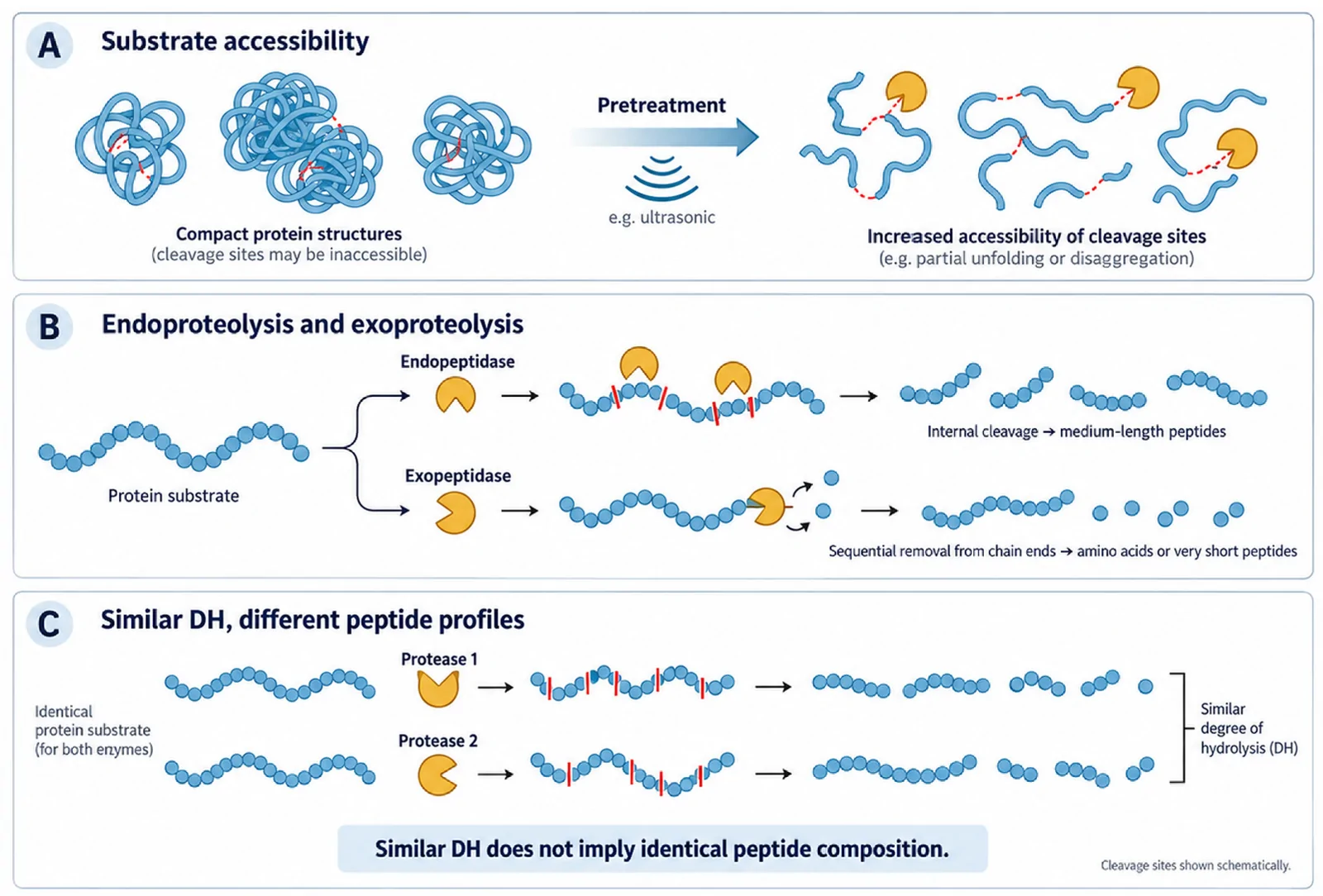
Substrate accessibility and protease specificity shape the peptide composition of fish protein hydrolysates. (**A**) Increased accessibility of cleavage sites following pretreatment; (**B**) peptide formation through endoproteolysis and exoproteolysis; (**C**) different peptide profiles obtained at similar degrees of hydrolysis (DH).

Commercial proteases differ in cleavage specificity and formulation. Alcalase commonly drives rapid endoproteolysis; Neutrase and Protamex generate different cleavage patterns; Flavourzyme combines endo- and exopeptidase activities; and papain, bromelain, pepsin and trypsin provide alternative specificities (Figure 2B). Because commercial and crude enzyme preparations differ in activity units and side activities, dosage should be expressed both as preparation mass and catalytic activity per gram of substrate protein under a defined assay. Accessibility of cleavage sites also depends on substrate structure and pretreatment (Figure 2A), and proteases that produce a similar DH may generate different peptide profiles (Figure 2C).

The importance of activity-equivalent comparison was demonstrated for Atlantic salmon by-products using Alcalase 2.4L (Novozymes, Bagsværd, Denmark), Corolase 7089 (AB Enzymes, Darmstadt, Germany), Neutrase 0.8L (Novozymes, Bagsværd, Denmark), Promod 671L (Biocatalysts, Cardiff, UK) and Protex 7L (DuPont, Wilmington, DE, USA) at 5.5–88 U g^−1^ protein. At low activity-to-substrate ratios, the enzymes showed broadly comparable cost efficiency, whereas at higher dosages Alcalase, Protex and Promod produced greater DH than Corolase and Neutrase. Nevertheless, additional peptide-bond cleavage did not result in a proportional increase in protein solubilisation, indicating that high enzyme dosages increasingly hydrolysed peptides already present in the soluble phase. In a separate sensory study, Alcalase-derived hydrolysates were more bitter than those produced with Promod or Protex, particularly at a DH above approximately 25% and with an increased proportion of peptides below 2 kDa [31].

Endogenous proteases provide a circular alternative to purchased enzymes, although activity normalization is especially important for crude extracts. Alkaline proteases extracted from fish viscera remained active principally at pH 8–9 and 40–50 °C. Hydrolysis with the crude visceral extract required an enzyme/substrate (E/S) ratio of 10% and 6 h, compared with 3% and 2 h for Alcalase. The resulting hydrolysates showed similar antioxidant performance in some chemical assays but differed in angiotensin-converting enzyme (ACE)-inhibitory activity. Thus, nominal percentages of crude extract and purified commercial enzymes are not directly comparable without measurements of catalytic activity, extract protein concentration and protease composition [32].

### 3.2. Enzyme/Substrate Ratio and Reaction Environment

The E/S ratio is reported on incompatible bases across studies, including wet material, dry matter, commercial-enzyme mass and catalytic activity. Comparisons are meaningful only when substrate protein mass and activity-based dosing are specified. Increasing E/S generally accelerates early hydrolysis but gives diminishing returns as accessible cleavage sites are depleted. In sardine by-products, a condition using 4.682% enzyme and approximately 26–31% solids produced 25.7% DH, 69.1% dry-solids yield and 83.0% protein recovery, illustrating that the DH and recovery must be optimized jointly [33].

pH, temperature, solids concentration and mixing jointly affect enzyme activity, protein solubility, heat transfer, phase separation and oxidation. Scale-up should therefore report temperature- and pH-time histories rather than set points alone because gradients increase with reactor volume. pH-stat control improves control of enzyme activity but adds alkali or acid, increasing salt, ash and processing cost. Uncontrolled pH drift can also be reproducible if the initial and final pH and the reaction profile are documented; the choice should therefore be justified by process objectives rather than treated as a universal requirement.

Salmon frames hydrolysed with Alcalase at 55 °C were compared at 50, 75 and 100% raw-material contents under three pH regimes: pH-stat control at pH 8, initial adjustment to pH 8 without subsequent control, and hydrolysis at the native pH of approximately 6.4. Decreasing the amount of added water increased the concentration of released α-amino groups, but nitrogen recovery decreased from approximately 50% at 50% raw material to 24% in the undiluted system. The water-to-raw-material ratio exerted a greater effect than the selected pH regime and shifted the estimated average peptide-chain length from approximately 5–7 to 7–9 residues [34]. Low-solids slurries generally facilitate mixing, heat transfer and phase separation, whereas high-solids operation reduces reactor volume, water use and subsequent evaporation requirements. These advantages cannot be evaluated from DH alone. In sardine hydrolysis, protein losses into emulsion and sludge phases varied markedly with solids concentration, enzyme dose and mixing. Under a selected condition, approximately 5.8% of the protein was retained in the emulsion and 11.2% in the sediment, while emulsion-associated losses across the experimental design ranged from approximately 1.5 to 15.5% [35].

Controlled endpoint design can also be based on a required molecular-weight range rather than a fixed hydrolysis time or maximum DH. Hydrolysis of tuna-canning residues and discarded blue whiting was modelled and validated at 5 L scale to produce peptide populations centred within 1–2, 2–5 and 5–10 kDa ranges, predominantly without continuous pH regulation. This approach linked operating conditions to a predefined molecular target and distinguished hydrolysates that would otherwise be described only by a global DH value [36].

### 3.3. Hydrolysis Endpoint, Enzyme Inactivation and DH Measurement

Protein extraction, peptide-bond cleavage and formation of a specific bioactive fraction are related but non-equivalent process responses. During cod-backbone hydrolysis, Alcalase produced a protein-extraction yield of 63.5% after 3 h, compared with 51.9–56.5% for Protamex and 30.4–34.7% for Neutrase. No consistent relationship was observed between DH and protein-extraction yield. Protamex hydrolysis for 6 h generated the strongest DPPH response, whereas the non-enzymatic heated control showed comparable or stronger iron-chelating activity. None of the hydrolysates exhibited the proposed antimicrobial activity [37]. A longer reaction or greater DH may therefore improve one measured function while leaving another unchanged or reducing it.

Enzyme inactivation defines the effective hydrolysis endpoint. Reported treatments range from 85 to 100 °C for 10–15 min, but heating rate, core holding time, cooling and residual activity determine whether proteolysis truly stops [16,38,39]. Alternative termination methods should likewise be verified by residual-activity testing.

DH is commonly calculated as:

$$\mathrm{DH}\left(\%\right)=\frac{h}{h_{\mathrm{tot}}}\times 100,$$

where *h* is the number of hydrolysed peptide bonds and *h_tot_* is the total number of peptide bonds in the substrate. The result depends on the analytical method and on the *h_tot_* value selected for the particular protein source.

The o-phthaldialdehyde (OPA) assay estimates newly formed primary amino groups and is relatively rapid but requires appropriate calibration, blanks and a justified substrate-specific *h_tot_* value [40]. The 2,4,6-trinitrobenzenesulfonic acid (TNBS) assay also measures amino groups, whereas pH-stat estimates cleavage from base consumption and the average dissociation state of released groups. Formol titration provides another operational estimate. By contrast, the proportion of nitrogen soluble in trichloroacetic acid describes soluble low-molecular-weight nitrogen and should not be reported as an interchangeable measure of true peptide-bond cleavage [41]. Direct comparison of DH values obtained by different methods can be misleading. In a controlled comparison of whey protein hydrolysates, OPA produced DH values approximately 15% lower than pH-stat for Alcalase hydrolysis, while agreement deteriorated further for enzyme preparations containing substantial exopeptidase activity [42]. Each FPH study should therefore identify the assay method, standard, sample dilution and preparation, blank correction, equation and *h_tot_*. Size-exclusion chromatography, free-amino-acid analysis and sequence-resolved peptidomics provide complementary information that DH alone cannot supply.

A comparison of representative studies demonstrates that DH is not determined by a single standardized analytical procedure. Aspevik et al. [43] used both pH-stat and o-phthaldialdehyde (OPA) measurements for Atlantic salmon by-products, reporting DH values of 11.6–14.6% after 120 min at the highest enzyme activity-to-substrate ratios. However, increased cleavage did not translate into a proportional increase in recovered soluble protein. By contrast, Borges et al. [32] applied the 2,4,6-trinitrobenzenesulfonic acid (TNBS) assay and reported approximately 40% DH after 6 h with crude fish-viscera proteases and approximately 50% after 2 h with Alcalase. These values cannot be used to rank enzyme efficiency directly because the enzyme preparations, doses, reaction times and DH measurement procedures differed.

Chiodza and Goosen [33] monitored DH by pH-stat titration in sardine by-products and obtained 25.7% DH together with 69.1% dry-solids yield and 83.0% protein recovery under their selected conditions; nevertheless, the significant lack of fit of the DH regression model limits the generalizability of its predictive relationships. Valencia et al. [34] used an OPA-based estimate of released soluble α-amino groups to calculate an approximate DH (DH′) in salmon frames. Their pH-stat regime was a process-control treatment, not the analytical basis of the reported DH′. Nitrogen recovery declined from approximately 50% to 24% when the fish-frame fraction increased from 50% to 100%, illustrating that greater process concentration may compromise recovery. In cod-backbone hydrolysates, Marinou et al. [37] used OPA and found no consistent relationship between DH and protein-extraction yield. These differences support the interpretation of DH alongside mass recovery and product properties rather than as a stand-alone processing endpoint.

Table 2 compares the DH determination methods and principal outcomes of these studies. Although pH-stat estimates bond cleavage from base consumption and OPA or TNBS quantify amino-group release, each approach depends on assumptions regarding the substrate and reaction environment. Approximate soluble-phase DH estimates introduce additional uncertainty. Consequently, numerical DH values should be compared across publications only when the analytical method, calibration, baseline correction, total peptide-bond content (*h_tot_*), protein substrate, and processing conditions are reported adequately. Greater DH does not necessarily imply improved protein recovery, peptide functionality or sensory acceptability.

### 3.4. Yield, Phase Separation and Membrane Fractionation

Dry-matter yield (Y_DM_) and protein recovery (PR) should be reported separately:

$$Y_{\mathrm{DM}}\left(\%\right)=\frac{\mathrm{dry matter recovered in the target product}}{\mathrm{dry matter in the initial feedstock}}\times 100,$$

$$\mathrm{PR}\left(\%\right)=\frac{\mathrm{protein recovered in the target product}}{\mathrm{protein in the initial feedstock}}\times 100.$$

Dry-matter yield, soluble-protein yield and total protein recovery are not interchangeable and should be reported for the same defined process stage. Carrier addition before drying can inflate apparent powder yield; carrier-free FPH recovery and protein recovery should therefore be stated separately.

Centrifugation and membrane processing redistribute protein among aqueous, lipid/emulsion and sediment fractions. Laboratory clarification can produce highly purified products, but pilot-scale separation may give lower recovery and different sensory outcomes. For example, pilot microfiltration and nanofiltration produced >90% protein/peptide retentates but reduced total protein yield to approximately 19–24%, while bitterness increased as small bitter peptides were retained [44].

Nominal membrane molecular-weight cut-off (MWCO) is not an exact peptide-size boundary because transmission also depends on membrane material, peptide charge and conformation, pressure, ionic strength and fouling. Low-molecular-weight fractions often show strong activity in some assays, but the optimum fraction is endpoint-specific: <3 kDa tuna-viscera permeate gave the strongest responses in several antioxidant and antibacterial assays, whereas >10 kDa tuna-cooking-juice fractions performed best in the 2,2′-azino-bis(3-ethylbenzothiazoline-6-sulfonic acid) (ABTS) and ferric-reducing assays [45,46].

Sequential Alcalase–Flavourzyme hydrolysis of tuna-head by-products to a final DH of approximately 18.5%, followed by ceramic filtration through 8, 3 and 1 kDa membranes, illustrates the integration of protease selection and membrane cut-offs to obtain application-oriented peptide ingredients [39]. Membrane studies should report membrane material and configuration, nominal MWCO, active area, transmembrane pressure, cross-flow velocity, temperature, volume-concentration factor, diafiltration volume, water flux, process flux, cleaning protocol and protein mass balance in every retentate and permeate.

### 3.5. Drying and Encapsulation

Freeze-drying is common at the laboratory scale, whereas spray drying is faster and more scalable. Their effects are condition-dependent rather than simply ‘mild’ versus ‘severe’. In bighead-carp skin hydrolysates, freeze- and spray-dried powders had similar protein contents (92.75 and 93.12%), while spray drying lowered moisture and increased some antioxidant responses without altering ABTS or ACE inhibition [47]. The minimum information required to reproduce each stage of controlled FPH production, from protease selection to encapsulation, is summarized in Table 3.

Encapsulation is a formulation, not purification, step. Wall materials such as maltodextrin, alginate and gum arabic can reduce hygroscopicity, oxidation, bitterness and fishy odour but dilute the peptide payload. Pink-perch FPH encapsulated with maltodextrin, sodium alginate and gum arabic achieved about 76% encapsulation efficiency and improved sensory properties; in a soup formulation, overall acceptability reached 7.6/9 [38,48,49]. Dose and bioactivity should therefore be expressed on both composite-powder and carrier-free FPH bases.

**Figure 3:**
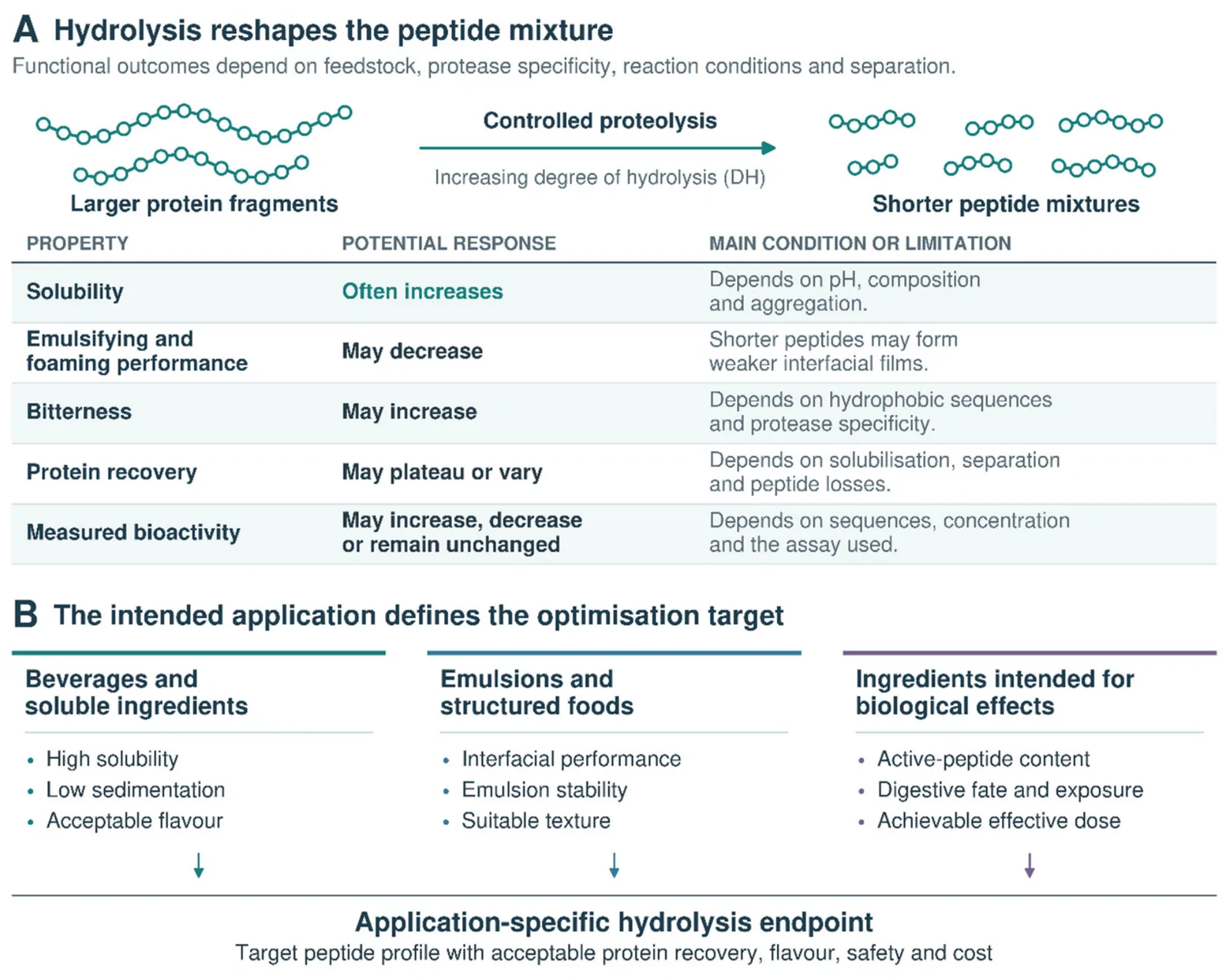
Application-dependent trade-offs in fish protein hydrolysate production. (**A**) Effects of increasing degree of hydrolysis (DH) on functional properties and bioactivity; (**B**) application-specific optimisation targets and selection of the hydrolysis endpoint.

Figure 3 shows that the preferred hydrolysis endpoint is application-specific: improvements in solubility or measured bioactivity can be accompanied by losses in interfacial performance, sensory quality, or protein recovery.

## 4. Peptidomics and Structure–Function Relationships

Peptidomics links controlled hydrolysis to peptide identity, but a liquid chromatography–tandem mass spectrometry (LC–MS/MS) list represents only the portion of the mixture recovered, chromatographically retained, ionized, selected and accepted by the search workflow. Analytical identification, computational prediction and experimental demonstration of function must therefore remain distinct.

### 4.1. LC–MS/MS Acquisition and Analytical Coverage

**Figure 4:**
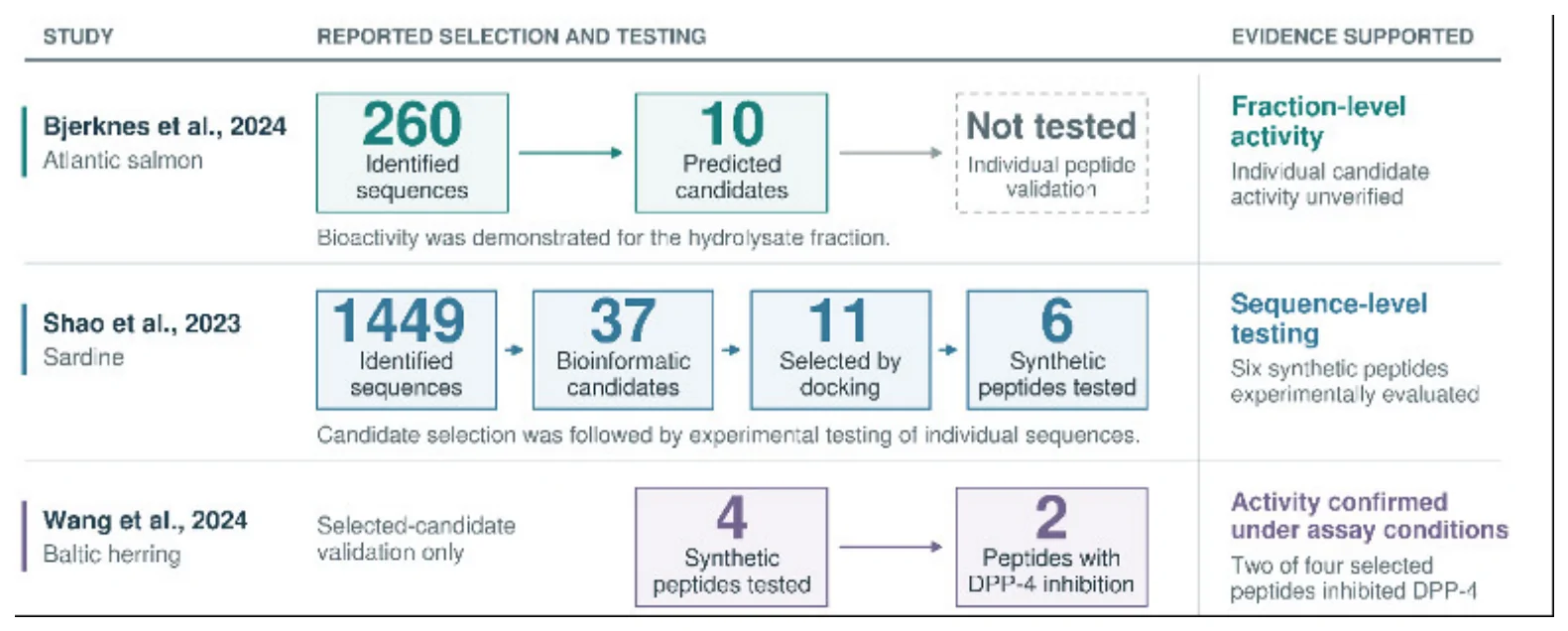
From peptide identification to experimental validation in representative FPH studies. DPP-4, dipeptidyl peptidase-4. Bjerknes et al. [50] reported fraction-level bioactivity without individual peptide validation; Shao et al. [52] performed sequence-level testing of selected synthetic peptides; Wang et al. [51] confirmed DPP-4 inhibitory activity in two of four tested peptides.

Peptidomic reporting should specify the mass spectrometer, liquid chromatography (LC) conditions, acquisition mode, fragmentation, mass ranges and resolution, quality-control strategy and injected amount. Data-dependent acquisition (DDA) preferentially fragments abundant precursors and is stochastic, whereas data-independent acquisition (DIA), including the MSE mode that alternates low- and elevated-collision-energy scans, samples broader precursor populations but requires more complex deconvolution. Consequently, peptide counts are method-dependent. This dependence is evident across recent studies. A Q Exactive top-12 DDA workflow with a 120 min gradient identified 260 sequences in a <3 kDa Atlantic salmon fraction [50], whereas an MSE/DIA quadrupole time-of-flight (QTOF) workflow reported 1150 and 985 peptides in Baltic-herring hydrolysates [51]. These numbers cannot be attributed to biology alone because acquisition, sample preparation, databases and search software differed. The progression from peptide identification to candidate prioritization and experimental validation in representative FPH studies is summarized in Figure 4.

Accordingly, peptide counts should be compared across studies only when sample fraction, LC gradient, acquisition mode, database, search engine, peptide-length limits, modifications and false-discovery rate (FDR) control are comparable. A larger peptide list does not by itself indicate more extensive or more selective hydrolysis.

### 4.2. Database Searching, De Novo Sequencing and False-Discovery Control

FPHs contain predominantly non-tryptic peptides with heterogeneous termini, so searches require nonspecific or permissive enzyme settings. This enlarges the search space and increases false-match risk; precursor and fragment tolerances, peptide length, charge states, modifications, database version and species coverage must therefore be reported. In Baltic herring, a nonspecific search against 37,476 *Clupea harengus* sequences used 10/20 ppm tolerances and 1% FDR, but the database still represented Atlantic rather than a dedicated Baltic-herring proteome [51].

De novo sequencing is useful when species proteomes are incomplete or unexpected cleavage products are present, but sequence confidence can be limited by incomplete fragmentation, spectral noise and unresolved Leu/Ile isomers. It should therefore be distinguished from database-supported identification, and confidence should be reported at residue and complete-sequence levels [53]. Database choice also requires scrutiny. In one eel-collagen study, spectra from *Anguilla japonica* were reportedly searched against a Pisum sativum database, making the parent-protein assignments difficult to interpret even though the synthesized peptides can be evaluated as separate chemical entities [54]. The FDR should be controlled at peptide–spectrum match and unique-peptide levels and, where proteins are inferred, at protein level. A 1% FDR is an expected error rate for the accepted set, not a 99% probability that every individual sequence is correct [55].

### 4.3. Parent Proteins, Unique Peptides and Peptidoforms

Parent-protein assignment can connect peptide profiles to feedstock anatomy, but abundant proteins and ionization bias also shape the observed spectrum. Assignments should therefore include accession, species, database version, peptide position and mapping uniqueness; shared collagen or myosin sequences should not be treated as exclusive markers of one parent protein. The term ‘unique peptide’ should be defined explicitly as a non-redundant sequence, a parent-protein-unique sequence, a peptide represented by one or more spectra, or a distinct modified peptidoform. Modification-aware reporting is especially important for collagen-derived FPHs because hydroxylation of proline changes mass, retention and potentially activity. A complete peptide table should therefore distinguish sequence and modified sequence and include mass, charge, retention time, mass error, identification confidence, spectral support, abundance, parent accession and mapping uniqueness.

### 4.4. Molecular Mass, Charge, Hydrophobicity and Sequence Arrangement

Peptide function cannot be inferred from molecular mass alone. Smaller peptides may access enzyme pockets more readily, but activity is not monotonic with size: in eel bone collagen, the 1–3 kDa fraction showed 79.33% ACE inhibition compared with 61.33% for the <1 kDa fraction [54]. Charge, hydrophobicity, residue order and terminal positions are target-dependent determinants of activity and sensory behaviour. Hydrophobic/aromatic residues not only may strengthen enzyme interactions but can also increase bitterness, reduce solubility and bias octadecyl (C18) reversed-phase chromatography and electrospray ionization (ESI) detection, while acidic or basic residues influence metal binding, taste and electrostatic interactions. Sequence order can be decisive even when composition and mass are identical. In sardine hydrolysate, synthetic KFL (Lys-Phe-Leu) and KLF (Lys-Leu-Phe) showed different activities despite containing the same residues, and dipeptidyl peptidase-4 (DPP-4) inhibition by salmon-gelatin peptides GPAE (Gly-Pro-Ala-Glu) and GPGA (Gly-Pro-Gly-Ala) illustrates the importance of N-terminal arrangement [52,56].

### 4.5. Predicted Activity Versus Experimental Validation

Bioinformatic classifiers, docking and molecular dynamics are candidate-screening tools, not evidence of sequence-level activity. In Atlantic salmon, ten of 260 identified peptides were prioritized computationally but not synthesized (Figure 4); the <3 kDa fraction inhibited DPP-4 and increased glucose uptake, validating the fraction rather than the predicted sequences [50]. In Baltic herring, only two of four synthesized candidates inhibited DPP-4, showing that prediction requires direct testing [51]. Sequence-level validation should answer two separate questions: whether the synthetic standard confirms the endogenous peptide identity by accurate mass, retention time and MS/MS fragmentation and whether the isolated peptide is active in a concentration–response assay [57]. Even then, activity does not prove that the peptide occurs in the original FPH at an effective concentration. Targeted quantification with isotopically labelled standards, followed by simulated digestion and LC–MS/MS confirmation of peptide persistence or daughter-peptide formation, provides the stronger translational link [58].

## 5. Evidence Ladder: From Assay Response to Human Efficacy

Biological evidence for FPHs should be interpreted as a progression from chemical reactivity to isolated-enzyme inhibition, cellular response, gastrointestinal stability, epithelial transport, animal physiology and randomized human trials. Each tier addresses a different question; higher tiers increase translational relevance but do not compensate for weak controls, inadequate exposure characterization or biassed outcome analysis.

### 5.1. Chemical Antioxidant Assays

DPPH, ABTS, ferric reducing antioxidant power (FRAP) and oxygen radical absorbance capacity (ORAC) assays probe different chemical mechanisms and should not be collapsed into a single ‘antioxidant activity’. Their results depend on solvent, pH, reaction time, peptide solubility, colour and turbidity [59]. Assay dependence is illustrated by tuna-cooking-juice fractions: <5 kDa material performed best in DPPH, >10 kDa material in ABTS and ferric reduction, and <5/5–10 kDa fractions in ferrous chelation [46]. Chemical assays therefore support only the specific reaction measured under the stated conditions, not antioxidant efficacy in cells, foods or humans [60].

### 5.2. Inhibition of Isolated Enzymes

ACE, DPP-4, α-glucosidase and α-amylase assays are more target-specific than radical tests but remain simplified biochemical systems. Percentage inhibition at one dose is weak evidence; concentration–response curves and half-maximal inhibitory concentration (IC_50_) values are preferable, and comparisons require matched enzyme source, substrate, buffer and normalization. Reported DPP-4 IC_50_ values, for example, range from about 0.08 mg/mL for salmon-gelatin hydrolysate to 2.12–2.90 mg protein/mL for industrial blue-whiting hydrolysates [61,62]. Synthetic-peptide testing provides stronger causal evidence than fraction-level activity but still does not establish in vivo exposure. In Baltic herring, two predicted peptides showed weak DPP-4 inhibition while two others were inactive, although the whole hydrolysate produced metabolic effects in mice [51].

### 5.3. Cellular Models and Mechanisms of Action

Cell models add uptake, membrane interaction and intracellular signalling to cell-free assays, but their relevance depends on cell type, exposure concentration, cytotoxicity, replication and mechanistic controls [63]. In murine STC-1 enteroendocrine cells, blue-whiting hydrolysates increased active glucagon-like peptide-1 (GLP-1) secretion and proglucagon production, with increased intracellular Ca^2+^ but not cyclic adenosine monophosphate (cAMP) [64]. This supports a calcium-associated response in that model, not a human endocrine effect. Likewise, salmon-hydrolysate receptor and pancreatic-cell responses were exploratory and did not consistently separate whole-mixture effects from concentration effects [65]. Mechanistic claims should therefore be supported by dose–response data, independent biological replicates, cytotoxicity testing and, where appropriate, receptor antagonists, pathway inhibitors or target-engagement experiments [66].

### 5.4. Simulated Gastrointestinal Digestion

Simulated gastrointestinal digestion tests whether an FPH is transformed during sequential gastric and intestinal exposure. The harmonized protocol developed by the INFOGEST international network on food digestion improves comparability by standardizing enzymes, electrolytes, bile, pH and phase duration [67]. Digestion effects are product-specific: salmon-gelatin hydrolysate retained ACE, DPP-4 and ORAC activities, whereas DPP-4 inhibition decreased in several industrial blue-whiting hydrolysates [62]. Low initial molecular weight therefore does not guarantee post-digestion stability. Unchanged post-digestion bioactivity does not prove that the original peptide survived because degradation products may replace its function. Digestion studies should therefore pair pre/post-peptidomics with activity normalized to recovered protein or peptide and include enzyme-only blanks. Digestion demonstrates transformation and bioaccessibility, not absorption.

### 5.5. Intestinal Transport and Caco-2 Models

Caco-2 transport models address a distinct question: whether peptides or metabolites can reach the basolateral compartment. Pacific hake hydrolysate retained antioxidant and ACE-inhibitory activity after simulated digestion and Caco-2 permeation, but the responsible transported species were not resolved [68]. Apparent permeability is calculated as P_app_ = (dQ/dt)/(A × C_0_), where Q is the amount of compound transported into the receiver compartment, t is time, A is the monolayer surface area and C_0_ is the initial donor concentration. Transport studies should report monolayer differentiation and integrity, donor concentration, sampling time, cytotoxicity and mass recovery and use LC–MS/MS to identify apical, cellular and basolateral sequences. Because Caco-2 models lack normal mucus, microbiota, motility, immune interactions and blood flow, basolateral recovery supports epithelial permeability but not human plasma exposure.

### 5.6. Animal Studies

Animal models integrate digestion, absorption and systemic physiology but add species-, disease- and dose-related confounding. Salmon-gelatin hydrolysate and a purified tuna-frame ACE-inhibitory peptide both reduced blood pressure in hypertensive rats, providing stronger in vivo support than isolated-enzyme assays, although plasma exposure and target engagement were not fully resolved [61,69]. Comparisons with intact protein are particularly informative. In obese Zucker rats, intact blue-whiting meal and Alcalase- or Protamex-derived hydrolysates similarly attenuated blood-pressure increases, so the effect could not be attributed specifically to hydrolysis [70]. Likewise, metabolic effects of whole Baltic-herring hydrolysate in mice cannot be assigned to the separately screened DPP-4 peptides [51]. Animal studies should therefore use matched protein/energy controls, justified doses, randomization and blinded outcome assessment and, where possible, quantify peptide exposure in intestinal contents, portal blood, plasma or target tissues [71].

### 5.7. Randomized Human Intervention Studies

Randomized controlled trials provide the most direct human evidence only when interpretation is based on the randomized comparison. Within-group improvement does not demonstrate efficacy unless the between-group difference in change is significant; crossover trials likewise require the within-participant contrast between interventions. Human findings remain product- and endpoint-specific. In a 90-day trial, 1.4 and 2.8 g/day of blue-whiting FPH produced greater changes in some body-composition measures and cholecystokinin (CCK)/GLP-1 than placebo, but all groups underwent caloric restriction and no clear dose–response was observed [72]. Acute cod-hydrolysate studies found lower postprandial insulin but no effect on glucose, GLP-1, ghrelin, satiety or fullness [73,74]. Chronic cod-hydrolysate supplementation (4 g/day for eight weeks) in adults with metabolic syndrome did not differ from placebo for glycemic markers, lipids, body composition or most satiety and inflammatory outcomes [75,76]. These two publications analyzed the same participants and should not be counted as independent replications. A four-week crossover sleep study showed within-phase improvements during fish-hydrolysate supplementation, but the primary intra-individual comparison with placebo was not significant [77]. Open-label and small pilot studies can generate hypotheses but require independent product-specific replication before efficacy claims are made. Human trials should prespecify a primary endpoint, justify sample size, report the randomized treatment estimate with confidence intervals, control multiplicity, document adherence and adverse events, and characterize the administered FPH batch, including feedstock, process, peptide-size distribution, dose, contaminants and oxidation status.

## 6. Technological and Sensory Properties

Technological performance depends on peptide size, charge, hydrophobicity and DH rather than protein content alone. Hydrolysis usually increases solubility but can weaken interfacial properties. Across marine FPHs, solubility reached 74.05–98.99%, while emulsifying capacity and stability varied widely [78]. In salmon-head and threadfin-bream hydrolysates, lower DH better preserved emulsifying and foaming properties even when higher DH increased selected in vitro activities [9,78,79]. Extensive hydrolysis also weakens gelation because short peptides cannot form the continuous networks produced by intact proteins. FPH therefore functions more often as a soluble co-ingredient than as the principal gelling agent. In texture-modified salmon products, FPH-containing formulations were softer than casein/whey formulations after heating and refrigerated storage [80,81].

Sensory quality reflects both peptide composition and residual non-protein components. Under comparable processing conditions, herring hydrolysates were more bitter and flavour-intense than salmon products and contained more peptides below 1 kDa, while fishy odour and colour deterioration were more closely linked to residual lipids, haem proteins and oxidation [16,31,82]. The acceptable dose is therefore matrix-specific. In pasta, 5% blue-whiting FPH gave the highest sensory scores, whereas 15% performed worst; in bread, only 1.5% met consumer preferences, while FPH in protein bars markedly reduced hardness but impaired sensory quality [83,84,85]. Debittering and flavour correction involve trade-offs. Membrane filtration can reduce ash, metabolites and fishy notes but may increase bitterness and decrease protein recovery, while adsorbents or β-cyclodextrin can remove hydrophobic compounds at the cost of selectivity and peptide loss [44,86,87]. Food-application studies should therefore report FPH dose on product and protein bases, matrix composition, processing and storage conditions, oxidation/colour markers, instrumental functionality and blinded sensory outcomes.

## 7. Safety

Safety cannot be inferred from edible raw material or from hydrolysis itself. Processing may remove, redistribute or concentrate hazards, so contaminants, biogenic amines, microorganisms, oxidation products, residual protease activity and allergens must be assessed in the finished FPH at the intended dose and through shelf life. Heavy-metal profiles vary with species, tissue and harvesting area. Some salmon, mackerel and hake hydrolysates contained low Hg, Cd and Pb, but Cd in mackerel products was a notable exception, showing that hydrolysis is not a decontamination step [19,88]. Representative final lots should be tested for Hg, Cd, Pb and As; when total arsenic is appreciable, inorganic-arsenic speciation is preferable [89]. Histamine and other biogenic amines mainly reflect microbial activity before or during processing and are not reliably destroyed by hydrolysis, heating, freezing or drying. This is particularly relevant to histidine-rich species such as tuna and mackerel; histamine should therefore be measured together with cadaverine, putrescine, tyramine and other relevant amines in raw material and final FPH [20,90].

FPHs should not be assumed to be sterile or intrinsically antimicrobial. Although one heat-treated salmon product remained microbiologically acceptable for 29 days at 4 °C, anchovy FPH supported Salmonella enterica survival during refrigerated storage [80,91]. Microbiological validation should include process lethality, post-process contamination control and end-of-shelf-life testing for relevant indicator and pathogenic organisms.

Oxidative stability is closely linked to raw-material freshness and residual lipids/haem pigments. Tilapia hydrolysates prepared from ice-stored fish showed higher peroxide value, TBARS, volatile compounds and fishy odour than products from fresh material, and hemoglobin accelerated oxidation without changing DH [82,92]. Both primary and secondary oxidation markers are therefore required. An inactivation temperature alone does not prove the absence of residual proteolysis. Residual activity should be measured after cooling, concentration and drying and, for liquid products, during storage because continued cleavage can alter peptide distribution, bitterness, functionality and allergen profiles [93,94,95]. Moisture, water activity, oxygen exposure and packaging barrier properties should also be specified. Parvalbumin is a major fish allergen, but tropomyosin and other proteins may also remain immunoreactive. Protease treatment can reduce detectable parvalbumin, but loss of a sodium dodecyl sulfate–polyacrylamide gel electrophoresis (SDS-PAGE) band or reduced signal in a generic enzyme-linked immunosorbent assay (ELISA) does not demonstrate loss of immunoglobulin E (IgE)-reactive epitopes [96,97]. Claims of reduced allergenicity require IgE-based assays and, where justified, effector-cell or clinical confirmation [24].

Batch reproducibility should be demonstrated with independently produced lots rather than analytical triplicates from one hydrolysis. Lot-release specifications should combine composition, DH by a consistent method, peptide-size or LC–MS fingerprints, contaminants, biogenic amines, oxidation, residual protease activity, microbiology, allergen measurements and sensory attributes within predefined application-specific limits.

## 8. Conclusions

FPHs are lot-defined ingredients whose composition and performance are determined by the entire production chain, from feedstock quality and preservation to hydrolysis, separation and formulation. Reproducible development therefore requires traceable raw materials, standardized characterization and confirmation across independent production batches.

Hydrolysis should be optimized for the intended application rather than for maximum DH. The appropriate endpoint is a defined peptide profile that balances recovery, techno-functional performance, sensory acceptability and cost.

Current biological evidence remains dominated by in vitro studies, while human findings are limited and product-specific. Peptidomic identification and computational prediction generate candidates; causal interpretation requires synthetic-peptide validation, quantitative confirmation in the hydrolysate, gastrointestinal and transport studies, and adequately powered randomized trials using characterized products and prespecified outcomes.

Translation into food or nutraceutical ingredients ultimately requires molecular reproducibility, matrix compatibility, consumer-acceptable dosing, safety and manufacturing feasibility. Fractionation or encapsulation should be judged against peptide retention and recovery, and safety must be established for the final formulation throughout storage. The decisive criterion is whether a reproducible FPH delivers its intended benefit at a safe, acceptable and economically feasible dose.

## Figures and Tables

**Table 1:** Minimum feedstock and pre-analytical information for FPH studies.

Domain	Required Information
Biological origin	Scientific name, wild or farmed origin, catch area or farm, harvest date and season
Anatomical composition	Fractions used, inclusion of gills, blood, liver or intestinal contents, and proportions in mixed streams
Cold chain	Time to chilling or freezing, material and storage temperatures, storage duration, packaging, thawing conditions, and freeze–thaw cycles
Baseline composition	Moisture, protein, lipid, and ash on wet- and dry-matter bases; analytical methods and nitrogen conversion factor
Initial quality	Primary and secondary lipid-oxidation markers; protein carbonyls; TMA and biogenic amines where relevant
Pretreatment	Washing, bleeding, mincing, demineralization, antioxidant addition, defatting method, and residual lipid
Lot traceability	Supplier, lot code, lot mass, pooling strategy, and number of independently collected batches

**Table 2:** Comparison of methods used to determine degree of hydrolysis and interpretation of selected FPH studies.

Study	DH Determination	Reported Outcomes	Critical Interpretation
Aspevik et al. [43]	pH-stat; OPA	DH 11.6–14.6% at the highest enzyme activity/substrate ratios (120 min).	Higher DH did not provide a proportional increase in protein recovery.
Borges et al. [32]	TNBS	DH ≈ 40% with crude visceral proteases (6 h); ≈50% with Alcalase (2 h).	Different enzyme formulations, dose and duration preclude direct efficiency ranking.
Chiodza and Goosen [33]	pH-stat	DH 25.7%, dry-solids yield 69.1% and protein recovery 83.0% under selected conditions.	A significant lack of fit limits the predictive DH model.
Valencia et al. [34]	OPA-based approximate DH′	Nitrogen recovery declined from ~50% to ~24% as fish-frame content increased.	DH′ is an approximation; pH-stat was used for reaction control, not DH′ calculation.
Marinou et al. [37]	OPA	Alcalase gave protein-extraction yield 63.5%; no consistent DH–recovery relationship.	The extent of cleavage did not reliably predict protein recovery.

**Table 3:** Minimum information required for reporting controlled FPH production.

Process Stage	Minimum Information
Protease	Enzyme name, biological source, supplier, batch, Enzyme Commission (EC) class, declared and measured activity; activity-assay substrate, pH, temperature and unit definition
Enzyme dosage	Mass of preparation and activity per gram of substrate protein; E/S basis clearly identified as wet material, dry matter or protein
Reaction system	Feedstock and water masses, total solids, batch volume, reactor scale, mixing regime, temperature–time and pH–time profiles
pH control	Initial and final pH; pH-stat reagent, concentration and consumption, or complete pH drift when uncontrolled
Endpoint and inactivation	Hydrolysis time, endpoint criterion, heating/cooling profile or alternative inactivation method, residual enzyme activity
DH and mass balance	DH method, calibration, *h_tot_*, blank and equation; dry-matter yield, soluble-protein yield and total protein recovery
Separation	Centrifugal force, time and temperature; masses and protein contents of aqueous, oil/emulsion and sediment phases
Membrane processing	Membrane material, configuration, area, MWCO, pressure, cross-flow velocity, temperature, concentration factor, flux and fraction-wise protein recovery
Drying	Drying method and complete operating conditions; powder recovery, protein, moisture and water activity
Encapsulation	Wall material, core-to-wall ratio, protein payload, encapsulation efficiency, release behaviour and sensory performance

## Data Availability

The original contributions presented in the study are included in the article; further inquiries can be directed to the corresponding authors.

## Abbreviations

The following abbreviations are used in this manuscript:

ABTS	2,2′-azino-bis(3-ethylbenzothiazoline-6-sulfonic acid)
ACE	angiotensin-converting enzyme
C18	octadecyl (reversed-phase chromatography)
Caco-2	human colorectal adenocarcinoma cell line
cAMP	cyclic adenosine monophosphate
CCK	cholecystokinin
DDA	data-dependent acquisition
DH	degree of hydrolysis
DIA	data-independent acquisition
DPP-4	dipeptidyl peptidase-4
DPPH	2,2-diphenyl-1-picrylhydrazyl
E/S	enzyme/substrate
EC	Enzyme Commission
ELISA	enzyme-linked immunosorbent assay
ESI	electrospray ionization
FDR	false-discovery rate
FPH(s)	fish protein hydrolysate(s)
FRAP	ferric reducing antioxidant power
GLP-1	glucagon-like peptide-1
IC_50_	half-maximal inhibitory concentration
IgE	immunoglobulin E
LC	liquid chromatography
LC–MS	liquid chromatography–mass spectrometry
LC–MS/MS	liquid chromatography–tandem mass spectrometry
MS/MS	tandem mass spectrometry
MSE	data-independent acquisition mode alternating low- and elevated-collision-energy scans
MWCO	molecular-weight cut-off
OPA	o-phthaldialdehyde
ORAC	oxygen radical absorbance capacity
PR	protein recovery
PV	peroxide value
QTOF	quadrupole time-of-flight
SDG	Sustainable Development Goal
SDS-PAGE	sodium dodecyl sulfate–polyacrylamide gel electrophoresis
TBARS	thiobarbituric acid reactive substances
TMA	trimethylamine
TNBS	2,4,6-trinitrobenzenesulfonic acid
Y_DM_	dry-matter yield
